# Urinary Metabolomic Profiling to Identify Potential Biomarkers for the Diagnosis of Behcet’s Disease by Gas Chromatography/Time-of-Flight−Mass Spectrometry

**DOI:** 10.3390/ijms18112309

**Published:** 2017-11-02

**Authors:** Joong Kyong Ahn, Jungyeon Kim, Jiwon Hwang, Juhwan Song, Kyoung Heon Kim, Hoon-Suk Cha

**Affiliations:** 1 Division of Rheumatology, Department of Internal Medicine, Kangbuk Samsung Hospital, Sungkyunkwan University School of Medicine, Seoul 03181, Korea; mdahnjk@skku.edu; 2 Department of Biotechnology, Graduate School, Korea University, Seoul 02841, Korea; kim131812@korea.ac.kr (J.K.); sjh5904@korea.ac.kr (J.S.); 3 Department of Internal Medicine, National Police Hospital, Seoul 05715, Korea; ninedw@empas.com; 4 Division of Rheumatology, Department of Medicine, Samsung Medical Center, Sungkyunkwan University School of Medicine, Seoul 06351, Korea

**Keywords:** Behcet’s disease, diagnosis, metabolomics, gas chromatography-mass spectrometry, biomarker, urine

## Abstract

Diagnosing Behcet’s disease (BD) is challenging because of the lack of a diagnostic biomarker. The purposes of this study were to investigate distinctive metabolic changes in urine samples of BD patients and to identify urinary metabolic biomarkers for diagnosis of BD using gas chromatography/time-of-flight–mass spectrometry (GC/TOF−MS). Metabolomic profiling of urine samples from 44 BD patients and 41 healthy controls (HC) were assessed using GC/TOF−MS, in conjunction with multivariate statistical analysis. A total of 110 urinary metabolites were identified. The urine metabolite profiles obtained from GC/TOF−MS analysis could distinguish BD patients from the HC group in the discovery set. The parameter values of the orthogonal partial least squared-discrimination analysis (OPLS-DA) model were *R*^2^*X* of 0.231, *R*^2^*Y* of 0.804, and *Q*^2^ of 0.598. A biomarker panel composed of guanine, pyrrole-2-carboxylate, 3-hydroxypyridine, mannose, l-citrulline, galactonate, isothreonate, sedoheptuloses, hypoxanthine, and gluconic acid lactone were selected and adequately validated as putative biomarkers of BD (sensitivity 96.7%, specificity 93.3%, area under the curve 0.974). OPLS-DA showed clear discrimination of BD and HC groups by a biomarker panel of ten metabolites in the independent set (accuracy 88%). We demonstrated characteristic urinary metabolic profiles and potential urinary metabolite biomarkers that have clinical value in the diagnosis of BD using GC/TOF−MS.

## 1. Introduction

Behcet’s disease (BD), also called Behcet’s syndrome, is a rare systemic vasculitis that is characterized by common symptoms of recurrent oro-genital ulceration, skin lesions, arthritis and uveitis [1,2,3]. The specific etiology of Behcet’s disease remains elusive. Considering the pathogenesis and symptoms of Behcet’s disease, it is a disease located between autoimmune and autoinflammatory diseases. Behcet’s disease is hard to diagnose because there are other illnesses that have similar symptoms to BD, and specific biomarkers to diagnose BD are still lacking. Potential candidate biomarkers for the diagnosis of BD, such as C-reactive protein (CRP) or HLA-51, have not achieved sufficient diagnostic sensitivity and specificity. To date, diagnosis of BD is only based on the clinical symptoms because no sensitive and specific relevant biologic tests are available. Because it may take months or even years for all the common symptoms to appear, the diagnosis of BD based on clinical criteria may not be made for long time. In view of these aspects, the marked importance of investigating new diagnostic biomarkers that allow reliable and early diagnosis of BD is highlighted. Efforts have been made to apply metabolomics to solve these diagnostic difficulties in the rheumatologic field [4].

Metabolomics is the large-scale study of small molecules, commonly known as metabolites, within cells, biofluids, tissue or organisms. Metabolomic analysis can be performed using easily accessible biofluids such as urine, blood or cerebrospinal fluid and may permit the identification of disease-specific metabolite signatures that may be useful as disease biomarkers. Several metabolomic studies of BD, rheumatoid arthritis (RA), or lupus nephritis have been performed using various analytical platforms and biologic fluids [5,6,7,8,9,10,11,12,13].

Urine is the by-product or waste fluid secreted by the kidneys. In mammals, urine serves as a means for flushing waste molecules collected from the blood, and for homeostasis of body fluids, thereby better reflecting the changes in human metabolism [14]. It has an advantage that the metabolite concentration is higher in urine than in human plasma or serum. Urine contains many metabolites that can know the imbalance of almost all biochemical pathways of the body. Moreover, compared to plasma, urine is more readily available and is noninvasively collected [15]. From these findings, urine is perhaps the ideal fluid for metabolomic analysis.

Here, a metabolomics approach using gas chromatography/time-of-flight–mass spectrometry (GC/TOF−MS) combined with multivariate data analysis was applied to investigate the global urinary metabolite profiles of BD patients and healthy controls (HC) with the aim of identifying useful diagnostic urinary metabolic biomarkers of BD. Also, we evaluated the feasibility of employing urine metabolomic biomarker panels for reliable diagnosis of BD.

## 2. Results

### 2.1. Identification of Metabolites in the Urine Samples of the Discovery Set

A total of 110 metabolites were consistently detected in the urine samples of the discovery set. Because intensity of urea is too high, resulting in misinterpretations of data, urea was removed and 109 metabolites were used in this study. Identified metabolites were classified into several chemical classes based on Medical Subject Headings (MeSH) Tree, consisting of organic acids (23.6%), amino acids (21.8%), sugars and sugar alcohols (21.8%), fatty acids (12.7%), amines (11.8%), phosphates (1.8%), and others (6.4%) (Table S1).

### 2.2. Distinct Metabolomic Profiles in Urine Samples of Behcet’s Disease Patients Using Discovery Set

Unsupervised principal component analysis (PCA) with 109 metabolites was carried out to obtain an overview of the urinary GC/TOF−MS data from BD patients and HC group. The PCA score plot using 2 axes was not able to completely distinguish between two groups, showing *R*^2^*X* of 0.311 and *Q*^2^ of 0.029, respectively (Figure S1).

**Figure 1:**
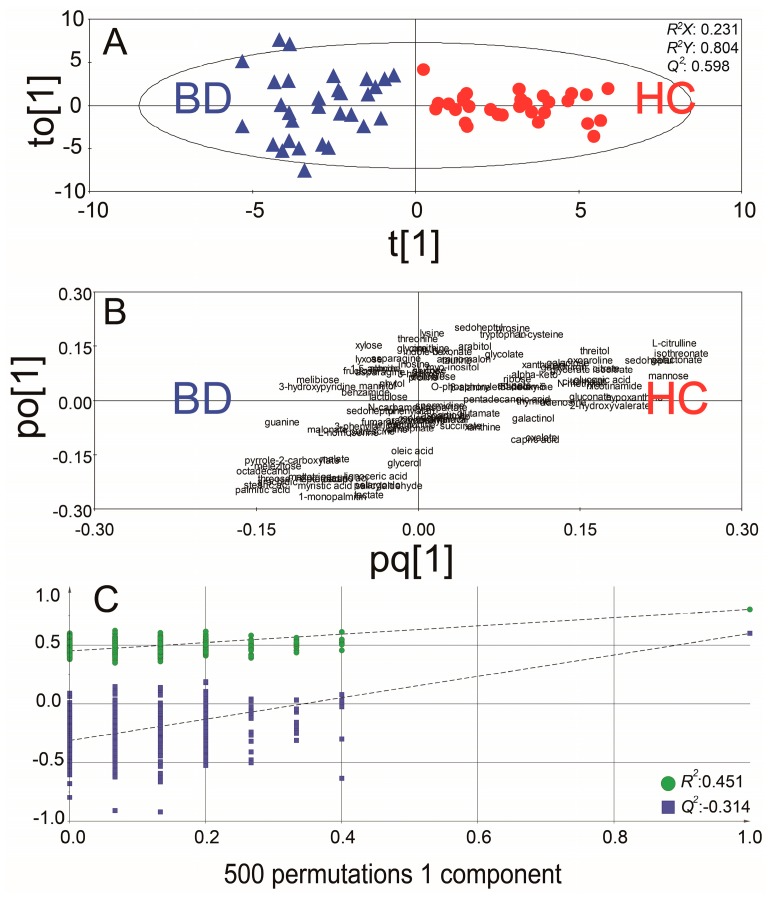
The score plot (**A**), and the loading plot (**B**) of the orthogonal partial least squared-discrimination analysis (OPLS-DA) using 109 identified metabolites of the discovery set. The OPLS-DA score plot showed clear separation between Behcet’s disease (*n* = 30) and healthy controls (*n* = 30) in the discovery set using 109 identified metabolites (*R*^2^*X*, *R*^2^*Y* and *Q*^2^, at 0.231, 0.804 and 0.598). In the permutation tests, the *Y*-axis intercepts of *R*^2^ and *Q*^2^ are 0.451 and −0.314, respectively, indicating that the original model is valid (**C**). The blue triangles and red circles denote Behcet’s disease (BD) and healthy controls (HC), respectively.

To capture the distinctive metabolic phenotypes and to maximize the discrimination between BD and HC groups by means of all the 109 identified urinary metabolites, we applied the orthogonal partial least squared-discrimination analysis (OPLS-DA). In an OPLS-DA score plot for the GC/MS data of the discovery set, a significant biochemical discrimination between BD patients and HC groups was identified with *R*^2^*X* of 0.231, *R*^2^*Y* of 0.804, and *Q*^2^ of 0.598, respectively (Figure 1A). The loading plot of OPLS-DA showed a brief overview of the contribution of each metabolite to the OPLS-DA model (Figure 1B and Table S2).

To prevent original model overfitting, model cross-validation through permutation tests with 500 iterations were performed (Figure 1C). These permutation tests produced intercepts of *R*^2^ and *Q*^2^ with values of 0.451 and −0.314. The criteria for validity were as follows: *R*^2^ less than 0.4 and *Q*^2^ less than 0.05, and all points of permuted *R*^2^ values should be located on the lower side in contrast to the original point. The PLS-DA model showed relatively high *R*^2^ value but met other criteria such as *Q*^2^ and location of points. Thus, the results indicate that the OPLS-DA models generated from GC/MS data were not over-fitted and reliable.

**Figure 2:**
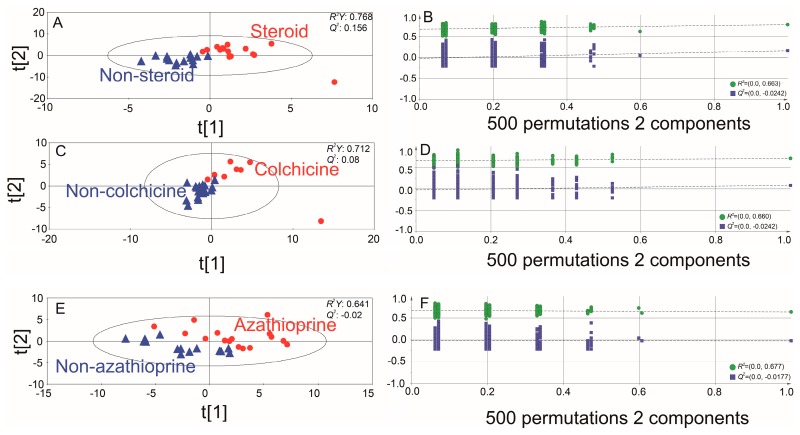
Score plots of the partial least square-discriminative analysis (PLS-DA) models (**A**,**C**,**E**) and 500 permutation tests using 2 components of the PLS-DA model (**B**,**D**,**F**). These models were generated according to the use of steroids, colchicine, and azathioprine in Behcet’s disease.

To evaluate the metabolic effects of medications used to treat patients with BD, the urine metabolic profiles of BD patients receiving medications (colchicine, azathioprine, or steroids) were compared with those not receiving these medications by partial least squares discriminant analysis (PLS-DA) analysis (Figure 2). Although all the PLS-DA models showed a slight discrimination tendency with R^2^Y from 0.641 to 0.768, there was no predictive capability: *Q*^2^ values from −0.02 to 0.156. Permutation tests with 500 iterations for the PLS-DA models revealed that many points of permutated *R*^2^ and *Q*^2^ values were located on the upper side in contrast to the original point.

### 2.3. Identification of Potential Biomarkers in the Discovery Set

Based on the OPLS-DA results, which facilitated a good separation of BD and HC groups, we extracted potential urinary biomarkers from a combination of variable importance on projection (VIP) value and statistical criteria including fold change, area under the receiver operating characteristic (ROC) curve (AUC), and *p* value. VIP values of all the metabolites from the OPLS-DA model were determined. Metabolites that have VIP values > 1.0, fold change > 1.5, AUC above 0.800 and *p* values below 0.01 were selected as potential biomarkers in our study. As a result, ten metabolites with discriminant potential between BD and HC—three amines derivatives (guanine, 3-hydroxypyroline, hypoxanthine), two amino acids derivatives (l-citrulline, isothreonate), three organic acids derivatives (pyrrole-2-carboxylate, galactonate, gluconic acid lactone), and two sugar and sugar alcohols (sedoheptulose, mannose)—were identified (Table 1). The abundance of guanine, pyrrole-2-carboxylate, and 3-hydroxypyroline was significantly higher in BD compared to HC, while hypoxanthine, l-citrulline, isothreonate, galactonate, gluconic acid lactone, sedoheptulose, and mannose was significantly lower in BD relative to BD.

### 2.4. The Multivariate Statistical Model with the Potential Urinary Biomarkers Selected for Robust Diagnosis of BD

**Figure 3:**
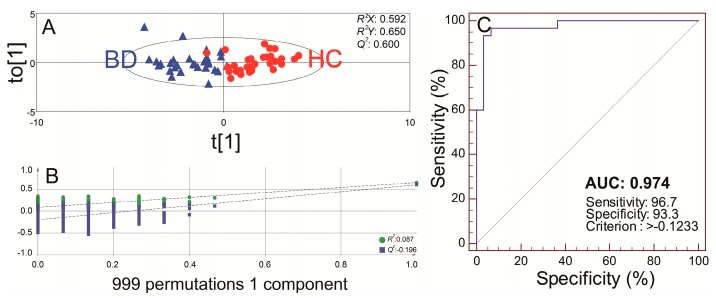
The score plot of the orthogonal partial least squared-discrimination analysis (OPLS-DA) (**A**), and a receiver-operating characteristic (ROC) curve of the model (**B**) in the discovery set based on 10 potential metabolic biomarkers. The numbers represent the sample numbers. In the OPLS-DA model, the blue triangles and red circles denote Behcet’s disease (BD) and healthy controls (HC), respectively. The ROC curve of the model had an AUC of 0.974 and high values of sensitivity (96.7%) and specificity (93.3%) (95% confidence interval 0.896–0.998) (**C**).

To create a robust diagnostic model for BD, another OPLS-DA was carried out to set up a simple statistical model using ten potential urinary biomarkers for the diagnosis of BD (Figure 3A). The OPLS-DA model using one component showed clear separation between groups BD and HC. Samples of the BD group showed a negative range of t[1] scores (−1.70 ± 1.37). In contrast, samples of the HC group showed a positive range of t[1] scores (1.70 ± 01.16). This model also yielded excellent values of explanatory and predictive capabilities (*R*^2^*X*, *R*^2^*Y* and *Q*^2^ at 0.592, 0.650 and 0.600, respectively), and permutation tests with 999 iterations for the OPLS-DA models also showed that the model is not over-fitted because the intercept of *R*^2^ is lower than 0.4 and the intercept of *Q*^2^ is lower than 0.05, and all points of permuted *R*^2^ values were located on the lower side in contrast to the original point (Figure 3B). Also, in the ROC curve analysis, the biomarker panel had an AUC value of 0.974 and excellent values of sensitivity (96.7%) and specificity (93.3%), at the best cut-off points (Figure 3C).

**Figure 4:**
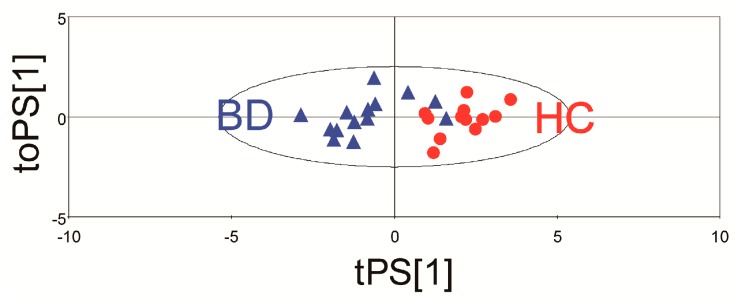
The score plot of the orthogonal partial least squared-discrimination analysis (OPLS-DA) in the independent set on the basis of 10 potential metabolic biomarkers. The blue triangles and red circles denote Behcet’s disease (BD) and healthy controls (HC), respectively.

To further evaluate the predictive ability of the established model, an independent test using urine samples from 14 patients and 11 controls was performed. The established model has a good potential to separate BD group from HC groups (Figure 4) and has a predictive ability with 88% accuracy.

### 2.5. Metabolic Pathway Analysis of BD

**Figure 5:**
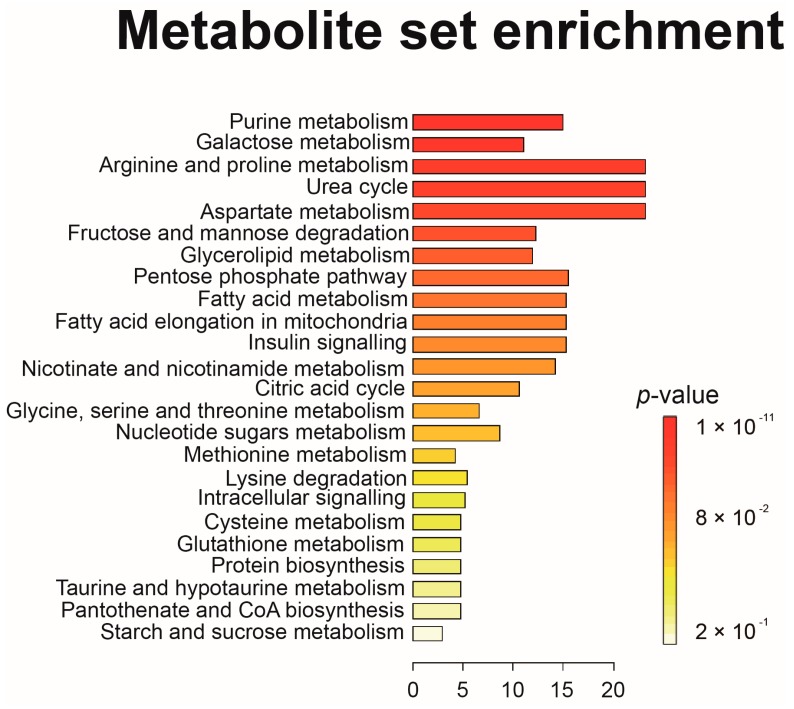
The impact of Behcet’s disease on serum metabolic pathways. To investigate whether metabolism is affected by BD, we performed pathway analysis using MetaboAnalyst. The *p* value and the pathway impact were calculated from the MSEA and the pathway topology analysis, respectively. The *p* value threshold was set to 0.01, and the values above this threshold were filtered out as insignificant pathways. The color codes of the bar plot correspond to the calculated *p* values. The color code of the bar plot corresponds to the calculated *p* values (red: *p* = 5 × 10^−6^ to white: *p* = 1.0).

We presented metabolite set enrichment analysis (MSEA) for the visualization and biological interpretation of metabolite data at the system level (Figure 5). MSEA demonstrated that the sugar metabolic pathways such as galactose metabolism, fructose and mannose degradation, and the pentose phosphate pathway; fatty acid metabolism and fatty acid in mitochondria; purine metabolism, arginine and proline metabolism, aspartate metabolism, nicotinate and nicotinamide metabolism, and glycerolipid metabolism were severely disturbed in BD group compared to HC groups.

## 3. Discussion

In the present study, metabolic profiles of patients with BD were established by using a GC/TOF–MS-based platform. To accomplish this, a total of 110 differential metabolites in the urine samples of BD and HC were identified on the result of GC/TOF−MS. We demonstrate the distinct urinary metabolic profiles of BD group compared to HC group. Based on statistical models, a potential urinary biomarker panel of 10 different metabolites (guanine, pyrrole-2-carboxylate, 3-hydroxypyroline, mannose, l-citrulline, galactonate, isothreonate, sedoheptulose, hypoxanthine, and gluconic acid lactonate) was screened for diagnosis of BD and appeared to have diagnostic value for BD with high sensitivity and specificity.

The diagnosis of BD is challenging because of a clinically driven diagnostic process and the lack of specific diagnostic biomarkers. Recently, metabolomics has been playing a significant role in identifying diagnostic biomarkers for various rheumatic diseases. Metabolomic studies using urine samples have been widely used to diagnose or predict treatment in a variety of rheumatic disease such as lupus nephritis, ankylosing spondylitis, or RA [7,8,9,10,11,12]. However, there are few metabolomic studies that investigate urine metabolites as diagnostic biomarkers of BD. Thus, we performed metabolic profiling to identify the potential urinary metabolic biomarker in patients with BD. In the present study using a GC/TOF−MS-based platform, we demonstrated the characteristic urinary metabolic profiles of BD group compared to HC group. Because the medication history of patients may influence metabolism, we tried to determine if there was a difference in urine metabolomic profile in BD depending on the medications use. As shown in Figure 2, there is no significant difference on metabolic profile according to mediation history, indicating that the effect of the drug on the urine metabolomic profiles is not significant in BD (the metabolic effect of medications does not seem to act as confounders and impact the classification of patients with BD). On the other hand, BD has the heterogeneous clinical manifestations. The fold change of each potential metabolic biomarker was statistically significant, but its level was low. Also, various factors affected fold changes of metabolic biomarkers in this study. In other words, several points may have altered the quantification results such as: (i) the dilution of the urine, (ii) the derivatization efficiency or (iii) matrix effect. Considering these findings: a biomarker panel consisting of multiple metabolites rather than a single biomarker may be a promising tool for making an effective diagnosis of BD; a biomarker panel consisting of multiple metabolites rather than a single biomarker may be a promising tool for making an effective diagnosis of BD. Based on several criteria, we have identified a set of 10 urinary metabolites contributing to the separation of BD from HC. In the OPLS-DA model, the *R*^2^ value represents the model ability to separate into classified groups while the *Q*^2^ value is a figure designed to represent the prediction capabilities of an unknown feature into the model. When using SIMCA-P+, *Q*^2^ value of more than 0.4 was considered as an acceptable biological model for use as a fully validated predicative tool. Thus, the parameters of *R*^2^*Y* of 0.804, and *Q*^2^ of 0.598 revealed the good discrimination and predictive ability of this model.

Behcet’s disease is characterized by vasculitis and endothelial cell dysfunction. It is known that increased nitric oxide (NO) production might be responsible for the overall inflammatory process and disease activity of BD [16,17]. Increased NO concentration in BD may play a role in endothelial activation, resulting in vascular inflammation and thrombosis. NO, an endothelium-derived relaxing factor, is a free oxygen radical that is produced by endothelial cells upon stimulation by immunologic, infectious, and inflammatory stimuli, such as TNF-α, or interferon-γ [3]. The activity of xanthine oxidase (XO) is increased in BD [18], which may increase free radicals and NO production. Hypoxanthine is oxidized successively to xanthine, and then to uric acid by XO. The abundance of hypoxanthine was significantly decreased in BD in this study. It is suggested that hypoxanthine may be used to produce free radicals and NO by increased XO activity in BD. On the other hands, the abundance of 3-hydroxypyridine, which has an anti-oxidant effect, was significantly increased in BD. The abundance of 3-hydroxypyridine may be increased due to a defense mechanism against oxidative stress such as NO.

Citrulline is produced from arginine as a byproduct of the reaction catalyzed by NOS family. Meanwhile citrulline is essential to make arginine, in turn, which is used to synthesize NO. Thus, citrulline would lead to boost NO production through arginine conversion [19]. In this study, patients with BD showed decreased abundance of l-citrulline compared with HC group, suggesting that it may have been exhausted for NO production. Measurement of urine citrulline in systemic lupus erythematosus is suggested to be a valuable adjunct to other tests as surrogate markers of NO production [20]. Citrulline may be used as a marker of disease activity as well as the diagnosis of BD. Taken together, the changes of hypoxanthine, 3-hydroxypyridine, and citrulline abundances in the urine suggest that they are the key metabolites of NO production, which may be responsible for oxidative tissue damage seen in BD. We have conducted a metabolomic study using synovial fluid to differentiate between BD with arthritis and seronegative arthritis [5]. Two biomarkers were identified, namely, methionine sulfoxide and citrulline, which were associated with increased oxidative stress. Also, increased oxidative stress in BD has been well documented. Considering these findings, potential metabolic markers related to oxidative tissue damage may offer opportunities for diagnosis of BD.

Galactonate is a product of alternative pathways of galactose metabolism, which is activated in MSEA. Galactonate is formed via oxidation of galactose by galactose dehydrogenase. Once formed, galactonate may enter the pentose phosphate pathway (PPP). The majority of immune cells participating in an inflammatory reaction shift their metabolism towards enhanced glucose uptake, aerobic glycolysis and increased activity of the PPP and fatty acid synthesis [13]. In this study, fatty acid metabolism and PPP are showed to be significant in MSEA, suggesting that immune cells that play an important role in BD may be activated.

Recent study reported that genera *Roseburia* and *Subdoligranulums*, which are key butyrate-producing members in intestine, in the stool of Behcet’s disease could reduce intestinal butyrate production [21]. Omics data integration of urine metabolomics and gut microbiota analysis may provide the powerful approach to discover novel metabolic biomarkers or investigate the mechanisms involved in BD.

Some limitations of this study need to be noted. First, the sample was limited in size in the context of biomarker discovery. Studies with a larger number of patients with BD are required to confirm this result to identify potential urinary metabolic biomarkers in BD. Second, neither we categorized BD patients into clinical subtypes due to small sample size nor we enrolled other rheumatic disease as positive control. Also, it may improve the completeness of this study to compare metabolomics profiles of intestinal BD with those of inflammatory bowel disease or to compare metabolomic profiles of BD with arthritis with those of seronegative arthritis. Additional research is necessary on a larger cohort of different manifestations of BD to diagnose clinical subtypes and to elucidate their pathogenesis. Also, further research including other autoimmune diseases or clinical mimics of BD is needed to determine whether our findings are specific to BD. Third, spot urine samples, which are usually collected at random, could not reflect the circadian rhythm of some metabolites. Fourth, urine metabolites are more complex than plasma metabolites. The collection of metabolite in the urine metabolite database is still far from complete. To facilitate future research into urine chemistry and urine metabolomics, it is critical to establish a comprehensive, electronically accessible database of the detectable metabolites in human urine [22]. Fifth, metabolites are sensitive to many environmental conditions including diet, drugs, physical exercise, gut microbiota, morbidities, hormonal status and age [4,23,24]. It is necessary to interpret the results of metabolomic studies considering that there are many uncontrollable factors in human studies.

This study demonstrates that a biomarker panel consisting of 10 potential urinary metabolites appeared to have diagnostic value for BD and deserve to be further validated. Additionally, this study suggests that GC/TOF−MS-based urine metabolomics is a promising screening tool to identify the biomarkers for diagnosis of BD. Also, these metabolomic results could help to understand the molecular mechanism involved in BD. Future research in this field will provide important information to monitor disease progression, predict prognosis, and guide treatment decisions.

## 4. Materials and Methods

### 4.1. Samples from Patients

Eighty-five samples were obtained from the rheumatology clinic at the Samsung Medical Center and Kangbuk Samsung Hospital in Seoul, Korea. Forty-four patients had BD and 41 healthy volunteers served as the HC group. The inclusion criteria for recruitment were as follows: aged > 18 years with independent right to sign a consent form with the criteria of the 1990 International Study Group for BD [25]. For all study participants enrolled as patients, the following demographic and clinical data at the time of blood sampling were obtained from the medical records and were analyzed: age at the time of diagnosis of BD, sex, duration of the disease, clinical signs and symptoms, medications and laboratory findings including erythrocyte sedimentation rate and CRP. Using a standard sterile procedure, urine samples were collected from all of the fasting volunteers in the morning and then centrifuged at 4 °C within 30 min. After that step, the supernatant was immediately frozen and stored at a temperature of −80 °C, until analyzed.

To create metabolic profiles, sixty urine samples from thirty patients with BD and age- and sex-matched thirty HCs were randomized into the discovery set. To validate the potential metabolic biomarker panel, twenty-five urine samples obtained from fourteen BD patients and eleven HCs were analyzed as the independent set. The baseline characteristics and clinical information of the participants are shown in Table S3.

The experimental protocols used in this study were approved by the Samsung Medical Center (#2014-01-082) and Kangbuk Samsung Hospital Institutional Review Board (#KBC14082), and written informed consent was obtained from each patient enrolled in this study. This study was conducted in accordance with the principles expressed in the Helsinki Declaration.

### 4.2. Metabolite Sample Preparation

Metabolites from urine were extracted using a previously reported procedure with a modification [26]. Each urine sample was thawed on ice for 60 min and vortexed for 1 min. An aliquot of 10 μL of urine was added into a labeled 1.5 mL micro-centrifuge tube containing 990 μL of pure methanol. The mixture was thoroughly vortexed for 5 min and then centrifuged at 16,100× *g* for 10 min at 4 °C. The supernatant was transferred into a 1.5 mL micro-centrifuge tube and completely dried using a vacuum concentrator (Labconco, Kansas City, MO, USA). The dried sample was derivatized in two steps. The sample was derivatized with 5 μL of 40 mg/mL methoxyamine hydrochloride in pyridine (Sigma-Aldrich, St. Louis, MO, USA) for 90 min at 30 °C for methoximation, and then with 45 μL of *N*-methyl-*N*-(trimethylsilyl) trifluoroacetamide (Fluka, Buchs, Switzerland) for 30 min at 37 °C for silylation. 2 μL of mixture of fatty acid methyl esters including methyl forms of C8, C9, C10, C12, C14, C16, C18, C20, C22, C24, C26, C28, and C30 was added to the derivatized sample as a retention index marker.

### 4.3. Quality Controls

For accurate analyses, daily quality control was exploited. Two method blanks (involving the reagents and the equipment used to control) and four calibration curve samples composed of 31 pure reference compounds including alanine, pyruvate, and various sugars were analyzed under the same analysis protocol. Intervention limits were set and laid out in a Standard Operating Procedure to ensure the validation of the instrument for metabolite profiling [27].

### 4.4. Identification of Metabolites and Data Processing

An Agilent 7890B Gas Chromatograph (Hewlett-Packard, Atlanta, GA, USA) coupled with a Pegasus HT TOF mass spectrometer (Leco, St. Joseph, MI, USA) was used for identification and relative quantification of the metabolites. An aliquot of 1.0 μL of the derivatized sample was injected into the gas chromatograph in a splitless mode. An RTX-5Sil MS capillary column (30 m length, 0.25 mm inner diameter, and 0.25 μm film thickness; Restek, Bellefonte, PA, USA) with an additional 10-m-long integrated guard column was used for separation of the metabolites. Initially, the oven temperature was set at 50 °C for 1 min, and then ramped to 330 °C at a rate of 20 °C/min and held at 330 °C for 5 min. Mass spectra were recorded in a mass range of 85–500 *m*/*z* at an acquisition rate of 10 spectra/s. The temperatures of the ion source and transfer line were set to 250 °C and 280 °C, respectively. The ionization was performed on electron impact at 70 eV.

### 4.5. Gas Chromatography-Time-of-Flight−Mass Spectrometry Data Processing and Statistical Analyses

For detection and deconvolution of the mass spectra, GC/TOF−MS data were pre-processed by the LECO Chroma TOF Software C version (Leco, St. Joseph, MI, USA). The pre-processed data were further processed using an in-house library, BinBase, for identification of metabolites. BinBase identified metabolites by comparing mass spectra and retention indices of peaks with the reference mass spectral and retention index libraries of the Fiehn library and NIST library, which were composed of over 1000 authentic standards [28]. The peaks meet retention time region of ±5 s and mass spectral similarity over 700 compared with the libraries were regarded as metabolites. Relative quantification of peaks was reported as peak height from the absolute unique ion intensity. A quantification table was produced for all database entries that were detected in more than 80% of the samples of the study design class. To process missing values, the mean of retention time was calculated and intensities of the quantification ions were subtracted by the lowest background intensity in a retention time region of ±5 s for each positively detected spectrum using the mzmine software [27]. The raw peak intensity of identified metabolites was normalized to median of the sum of the peak intensity of all the identified metabolites in each sample.

### 4.6. Statistical Analyses and Model Validation

To analyze the metabolite profiles of urine samples of the BD group and the HC group, multivariate statistical analyses including PCA, PLS-DA, OPLS-DA, univariate statistical analyses including Student’s *t* test and ROC curve analysis, and MSEA were performed. To obtain maximal covariance between the classes, PCA, PLS-DA, OPLS-DA, and a permutation test as model validation were carried out in the SIMCA-P+ software (version 12.0; Umetrics AB, Umea, Sweden). Student’s *t* test was performed using the Statistica software (version 7.1; StatSoft, Tulsa, OK, USA). The ROC curve analysis was conducted using the MedCalc software (Broek-straat, Mariakerke, Belgium). MSEA was performed using the Web-based MetaboAnalyst (http://www.metaboanalyst.ca) [29].

## Figures and Tables

**Table 1:** VIP, fold change, AUC, and *p* values of urine metabolites to be used as potential biomarkers for discrimination of patients with Behcet’s disease from healthy controls.

Metabolite	VIP	Fold	AUC	*p* Value
Metabolites with higher abundances in the BD group than in the control group				
Guanine	1.63	2.33	0.834	3.68 × 10^−5^
Pyrrole-2-carboxylate	1.40	1.95	0.806	1.01 × 10^−4^
3-hydroxypyridine	1.36	2.25	0.846	2.75 × 10^−3^
Metabolites with higher abundances in the control group than in the BD group				
Mannose	2.02	3.28	0.860	1.11 × 10^−8^
l-citrulline	1.87	2.08	0.884	2.19 × 10^−8^
Galactonate	1.79	1.78	0.856	1.54 × 10^−7^
Isothreonate	1.79	1.76	0.862	1.38 × 10^−7^
Sedoheptulose	1.55	1.69	0.820	9.53 × 10^−6^
Hypoxanthine	1.48	2.25	0.849	1.06 × 10^−4^
Gluconic acid lactone	1.24	2.07	0.818	8.33 × 10^−4^

AUC, area under the ROC curve; BD, Behcet’s disease; VIP, variable importance on projection.

## Supplementary Materials

Supplementary materials can be found at www.mdpi.com/1422-0067/18/11/2309/s1.

## Abbreviations

AUC	Area under the receiver operating characteristic curve
BD	Behcet’s disease
CRP	C-reactive protein
GC/TOF−MS	Gas chromatography-time-of-flight–mass spectrometry
HC	Healthy control
MSEA	Metabolite set enrichment analysis
NO	Nitric oxide
OPLS-DA	Orthogonal partial least squared-discrimination analysis
PCA	Principal component analysis
PLS-DA	Partial least squares discriminant analysis
PPP	Pentose phosphate pathway
RA	Rheumatoid arthritis
ROC	Receiver operating characteristic
VIP	Variable importance on projection
XO	Xanthine oxidase
AUC	Area under the receiver operating characteristic curve
