# Supplementary material for: Urinary Metabolomic Profiling to Identify Potential Biomarkers for the Diagnosis of Behcet’s Disease by Gas Chromatography/Time-of-Flight−Mass Spectrometry
Source: Int J Mol Sci. 2017 Nov 2;18(11):2309. doi: 10.3390/ijms18112309 (PMC5713278; doi:10.3390/ijms18112309)
Supplement: Supplementary file 1 [file ijms-18-02309-s001.docx]

**
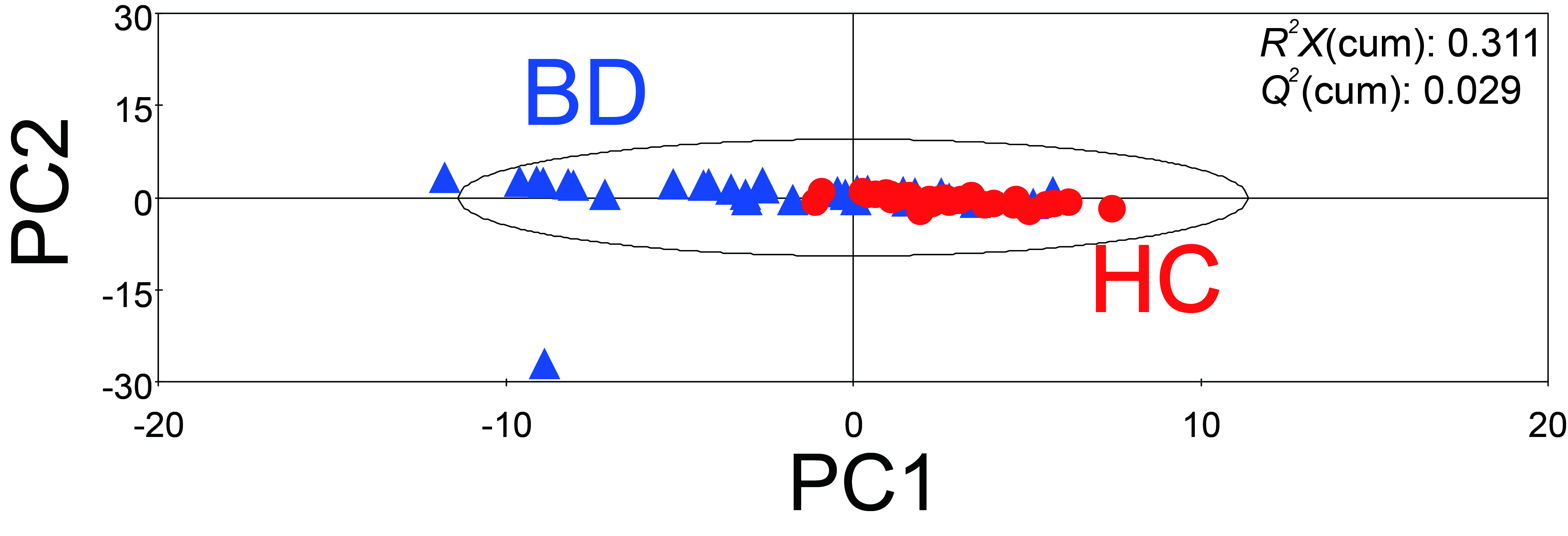
**

**Figure S1.** The score plot of the principal component analysis (PCA) of metabolomic profiles of urine samples from patients with Behcet’s disease (BD) and healthy controls (HC) in the discovery set. The blue triangles and red circles denote Behcet’s disease and healthy controls, respectively.

**Table S1.** Identification of 109 metabolites using BinBase in urine samples of patients with Behcet’s disease and healthy controls

| **Amines** |  |  |
| --- | --- | --- |
| 3-hydroxypyridine | 5'-deoxy-5'-methylthioadenosine | adenosine |
| benzamide | guanine | hypoxanthine |
| inosine | *O*-phosphorylethanolamine | putrescine |
| spermidine | thymine | xanthine |
| **Amino acids** |  |  |
| 5-aminovalerate | alanine | asparagine |
| asparagine dehydrated | glutamate | glycine |
| isoleucine | isothreonate | *L*-citrulline |
| *L*-cysteine | *L*-homoserine | lysine |
| methionine | *N*-methylalanine | ornithine |
| oxoproline | phenylalanine | proline |
| serine | threonine | tryptophan |
| tyrosine | valine | *β*-alanine |
| **Fatty acids** |  |  |
| 1-monopalmitin | arachidic acid | arachidonic acid |
| capric acid | heptadecanoic acid | lauric acid |
| lignoceric acid | myristic acid | octadecanol |
| oleic acid | palmitic acid | pelargonic acid |
| pentadecanoic acid | stearic acid |  |
| **Organic acids** |  |  |
| 2-hydroxyvalerate | 2-ketoadipate | 3-hydroxypropionate |
| 3-phenyllactate | adipate | aminomalonate |
| aspartate | citramalate | citrate |
| fumarate | galactonate | galacturonate |
| gluconate | gluconic acid lactone | glycerate |
| glycolate | hexonate | isocitrate |
| lactate | malate | malonate |
| *N*-carbamoylaspartate | oxalate | pyrrole-2-carboxylate |
| succinate | *α*-ketoglutarate |  |
| **Sugars and sugar alcohols** |  |  |
| 1,5-anhydroglucitol | arabitol | cellobiose |
| fructose | galactinol | galactose |
| glucose | glycerol | lactulose |
| lyxose | maltotriose | mannitol |
| mannose | melezitose | melibiose |
| myo-inositol | palatinitol | phytol |
| ribose | sedoheptulose | sedoheptulose anhydrous |
| threitol | threose | xylose |
| **Phosphates** |  |  |
| phosphate | sedoheptulose-7-phopsphate |  |
| **Others** |  |  |
| indole-3-lactate | nicotinamide | salicylaldehyde |
| taurine | uracil | uric acid |
| xanthurenic acid |  |  |

**Table S2.** bsolute loading values of the metabolites obtained from the orthogonal partial least squared-discrimination analysis using 109 metabolites.

| Var ID (Primary) | t[1] | to[1] |
| --- | --- | --- |
| 1,5-anhydroglucitol | -0.06609 | 0.09601 |
| 1-monopalmitin | -0.11401 | -0.25745 |
| 2-hydroxypyridine | -0.02137 | -0.04563 |
| 2-hydroxyvalerate | 0.13735 | -0.00629 |
| 2-ketoadipate | -0.11147 | -0.20807 |
| 3-hydroxypropionate | -0.02184 | 0.08047 |
| 3-hydroxypyridine | -0.13243 | 0.04675 |
| 3-phenyllactate | -0.08093 | -0.06332 |
| 5-aminovalerate | -0.00170 | -0.04379 |
| 5'-deoxy-5'-methylthioadenosine | 0.07310 | 0.04650 |
| adenosine | 0.11012 | 0.00287 |
| adipate | 0.07066 | 0.04897 |
| alanine | -0.04844 | 0.09570 |
| alpha-keto glutarate | 0.08339 | 0.07863 |
| aminomalonate | 0.01434 | 0.12192 |
| arabitol | 0.03407 | 0.15821 |
| arachidic acid | -0.15239 | -0.21724 |
| arachidonic acid | -0.03303 | -0.04606 |
| asparagine | -0.04677 | 0.12491 |
| asparagine dehydrated | -0.06122 | 0.08688 |
| aspartic acid | -0.00023 | -0.03133 |
| benzamide | -0.07448 | 0.03028 |
| capric acid | 0.08276 | -0.10048 |
| cellobiose | -0.00976 | 0.07426 |
| citramalate | 0.13035 | 0.05957 |
| citrate | 0.15885 | 0.09631 |
| fructose | -0.07269 | 0.08872 |
| fumarate | -0.05523 | -0.05204 |
| galactinol | 0.08392 | -0.04005 |
| galactonate | 0.21290 | 0.12108 |
| galactose | 0.11586 | 0.11216 |
| galacturonate | 0.11074 | 0.09943 |
| gluconate | 0.13668 | 0.01765 |
| gluconic acid lactone | 0.14096 | 0.06547 |
| glucose | -0.00854 | 0.09174 |
| glutamate | 0.03328 | -0.02775 |
| glycerate | 0.11952 | 0.09226 |
| glycerol | -0.03141 | -0.16426 |
| glycine | -0.02275 | 0.15191 |
| glycolate | 0.05884 | 0.13630 |
| guanine | -0.14523 | -0.05173 |
| heptadecanoic acid | -0.11655 | -0.20740 |
| hexonate | 0.01037 | 0.14289 |
| hypoxanthine | 0.17116 | 0.01690 |
| indole-3-lactate | -0.01662 | 0.14383 |
| inosine | -0.02118 | 0.10949 |
| isocitrate | 0.15887 | 0.09351 |
| isoleucine | -0.01985 | -0.04170 |
| isothreonate | 0.21529 | 0.13762 |
| lactate | -0.06187 | -0.25249 |
| lactulose | -0.04863 | 0.01775 |
| lauric acid | -0.08515 | -0.20663 |
| *L*-citrulline | 0.21369 | 0.16597 |
| *L*-cysteine | 0.08999 | 0.18961 |
| *L*-homoserine | -0.09605 | -0.08136 |
| lignoceric acid | -0.07157 | -0.20084 |
| lysine | -0.00142 | 0.19372 |
| lyxose | -0.06144 | 0.12346 |
| malate | -0.09425 | -0.15414 |
| malonate | -0.10575 | -0.07156 |
| maltotriose | -0.12350 | -0.20272 |
| mannitol | -0.05785 | 0.04701 |
| mannose | 0.21000 | 0.07490 |
| melezitose | -0.15504 | -0.17352 |
| melibiose | -0.11607 | 0.06544 |
| methionine | -0.03125 | -0.05755 |
| myo-inositol | 0.00412 | 0.10062 |
| myristic acid | -0.11491 | -0.22666 |
| *N*-carbamoylaspartate | -0.04980 | -0.01136 |
| nicotinamide | 0.15292 | 0.04701 |
| *N*-methylalanine | 0.12560 | 0.05434 |
| octadecanol | -0.17179 | -0.18681 |
| oleic acid | -0.02810 | -0.13160 |
| *O*-phosphorylethanolamine | 0.00909 | 0.04617 |
| ornithine | -0.00865 | 0.15209 |
| oxalate | 0.09655 | -0.09419 |
| oxoproline | 0.13535 | 0.11924 |
| palatinitol | 0.00827 | -0.03556 |
| palmitic acid | -0.17263 | -0.23954 |
| pelargonic acid | -0.06268 | -0.22442 |
| pentadecanoic acid | 0.03919 | 0.01211 |
| phenylalanine | -0.03063 | -0.01994 |
| phosphate | -0.03069 | -0.06858 |
| phytol | -0.03892 | 0.05523 |
| proline | -0.01186 | 0.07291 |
| putrescine | -0.06673 | -0.07669 |
| pyrrole-2-carboxylate | -0.16405 | -0.15686 |
| ribose | 0.07617 | 0.06743 |
| salicylaldehyde | -0.06130 | -0.22705 |
| sedoheptulose | 0.18867 | 0.11993 |
| sedoheptulose anhydrous | 0.03065 | 0.20967 |
| sedoheptulose-7-phopsphate | -0.06978 | -0.02082 |
| serine | -0.00798 | 0.08918 |
| spermidine | -0.00551 | -0.00561 |
| stearic acid | -0.16426 | -0.22469 |
| succinate | 0.01757 | -0.06116 |
| taurine | 0.01926 | 0.11851 |
| threitol | 0.14663 | 0.14565 |
| threonine | -0.02208 | 0.17753 |
| threose | -0.15153 | -0.20720 |
| thymine | 0.08820 | 0.00604 |
| tryptophan | 0.05443 | 0.18974 |
| tyrosine | 0.06906 | 0.20701 |
| uracil | 0.01961 | -0.04427 |
| uric acid | -0.04328 | -0.05956 |
| valine | -0.03613 | -0.07145 |
| xanthine | 0.04026 | -0.06558 |
| xanthurenic acid | 0.09298 | 0.10748 |
| xylose | -0.06108 | 0.16106 |
| *β*-alanine | 0.02647 | 0.04445 |

**Table S3.** Demographic and clinical chemistry characteristics of patients with Behcet’s disease and controls in the discovery set and independent set

|  | **Discovery set** | | **Independent set** | |
| --- | --- | --- | --- | --- |
|  | **BD (*n* = 30)** | **HC (*n* = 30)** | **BD (*n* = 14)** | **HC (*n* = 11)** |
| Males (%) | 15 (50) | 15 (50) | 11 (78.6) | 7 (63.6) |
| Age | 48.47 ± 9.46 | 49.60 ± 9.10 | 45.57 ± 10.05 | 39.91 ± 5.96 |
| Symptoms & Signs |  |  |  |  |
| Recurrent oral ulcer | 30 (100) |  | 14 (100) |  |
| Genital ulcer | 21 (70.0) |  | 8 (57.1) |  |
| Ocular involvement | 4 (13.3) |  | 1 (7.1) |  |
| Positive Pathergy test | 4/13 (30.8) |  | 2/5 (40.0) |  |
| Vascular involvement | 8 (26.7) |  | 6 (42.9) |  |
| EN-like lesions | 17 (56.7) |  | 10 (71.4) |  |
| GI involvement | 7 (23.3) |  | 2 (14.3) |  |
| CNS involvement | 2 (6.7) |  | 2 (14.3) |  |
| Laboratory findings |  |  |  |  |
| WBC (× 10^3^/mm^3^) | 7.06 ± 2.71 |  | 6.42 ± 1.67 |  |
| Hb (g/dl) | 13.61 ± 1.40 |  | 14.41 ± 1.71 |  |
| Platelet (× 10^3^/mm^3^) | 226.28 ± 49.39 |  | 226.93 ± 51.29 |  |
| Globulin (g/dl) | 2.79 ± 0.32 |  | 2.93 ± 0.48 |  |
| ALT (IU/l) | 20.34 ± 10.06 |  | 23.29 ± 15.54 |  |
| Cr (mg/dl) | 0.91 ± 0.39 |  | 0.86 ± 0.14 |  |
| ESR (mm/h) | 15.34 ± 11.47 |  | 23.43 ± 19.52 |  |
| CRP (mg/dl) | 0.36 ± 0.61 |  | 0.41 ± 0.57 |  |
| HLA-B51 | 4 /12 (33.3) |  | 5/9 (55.6) |  |
| Medications |  |  |  |  |
| Steroid | 14 (46.7) |  | 4 (28.6) |  |
| NSAID | 5 (16.7) |  | 1 (7.1) |  |
| Colchicine | 22 (73.3) |  | 9 (64.3) |  |
| Azathioprin | 14 (46.7) |  | 5 (35.7) |  |
| Sulfasalazine | 6 (20.0) |  | 0 (0) |  |
| Cyclosporine | 0 (0) |  | 1 (7.1) |  |
| Methotrexate | 1 (3.3) |  | 0 (0) |  |

Continuous variables are expressed as mean ± standard deviation (SD). Categorical variables are described as numbers and percentages. We recorded subject numbers with available clinical parameters.

ALT: alanine aminotransferase; BD, Behcet’s disease; CNS: central nervous system; CRP: C-reactive protein; EN: erythema nodosum; ESR: erythrocyte sedimentation rate; GI: gastrointestinal; Hb: haemoglobin; HC, healthy control; HLA: human leukocyte antigen; NSAID: nonsteroidal anti-inflammatory drug; WBC: white blood cell count.
